# Experimental In Vivo Toxicity Models for Alcohol Toxicity

**DOI:** 10.5152/eurasianjmed.2023.23345

**Published:** 2023-12-01

**Authors:** Fatma Betül Yoladi, Edanur Burmaoğlu, Şaziye Sezin Palabiyik-Yücelik

**Affiliations:** 1 Department of Pharmaceutical Toxicology, Atatürk University Faculty of Pharmacy, Erzurum, Turkey; 2 Department of Pharmaceutical Toxicology, Hacettepe University Faculty of Pharmacy, Ankara, Turkey; 3 Atatürk University Faculty of Pharmacy, Erzurum, Turkey; 4 Clinical Research, Development and Design Application and Research Center, Atatürk University, Erzurum, Turkey

**Keywords:** Alcohol, experimental animal model, hepatotoxicity

## Abstract

Alcohol consumption poses a significant risk for the development of chronic illnesses, one of the leading causes of “preventable” disease and death worldwide. Harmful consumption of alcohol is thought to result in approximately 2.5-3 million deaths each year, the majority of which are caused by alcohol-related liver diseases. Hepatocellular carcinoma, cirrhosis, fibrosis, steatosis, and steatohepatitis are among the liver illnesses caused by alcohol. The mechanisms behind human diseases are often mimicked and understood through the use of animal models. Rodents are the ideal animals to study alcohol-related liver diseases. In these experimental models using rodents, the ethanol ratio, method of administration, and diet to be applied vary. Within the scope of this review, it is aimed at providing information about the experimental models used today for alcohol toxicity and the advantages and disadvantages of these models.

## Introduction

Ethanol has a sedative–hypnotic effect and is widely consumed throughout the world.^1,2^ Acute and chronic alcohol consumption is a significant risk factor for chronic diseases and is one of the fifth leading global causes of preventable disease and death. It can also cause addiction when consumed unconsciously. Harmful use of alcohol is thought to result in approximately 2.5-3 million deaths each year, the majority of which are caused by alcohol-related liver diseases.^3-5^ Alcohol consumption also has effects on the economies of countries.^1^ Although the toxic effects of alcohol affect multiple organs,^6,7^ the liver, the primary site of its metabolism, is the main target organ.^8-10^ Ethanol is metabolized in hepatocytes to acetaldehyde by the cytosolic alcohol dehydrogenase enzyme and to acetate by mitochondrial acetaldehyde dehydrogenase.^10,11^ Acetaldehyde, known for its hepatotoxic effect, is considered one of the main causes of alcohol-related liver diseases because it can cause functional disorders of key proteins by nonenzymatically binding to free amino groups in the proteins of liver cells.^11^ In addition to these direct effects on hepatocytes, activation of liver cells and hepatic stellate cells contributes to fibrosis and cirrhosis.^12^ Therefore, alcohol-related liver diseases manifest as a series of clinical disorders ranging from steatosis (fatty liver) to alcoholic hepatitis and can progress to more serious conditions such as fibrosis, cirrhosis, and hepatocellular carcinoma.^13-15^ Currently, there is no effective treatment accepted by the U.S. Food and Drug Administration for the toxicity of alcohol in humans.^13,14^ Therefore, the discovery of new therapeutic strategies is an important need for these patients.^16^ Although there are in vitro studies conducted to evaluate alcohol toxicity and the underlying mechanisms,^17^ in vivo models are more important to understand the effects in humans because it causes toxicity through metabolic activation. Also, alcohol is used as an agent to induce different organ toxicities for the experimental studies.^6,7,18,19^ Understanding the molecular mechanisms by which alcohol initiates and progresses liver damage may help find new and effective treatments. Therefore, experimental animal models that mimic alcohol-induced liver damage in humans are a necessity to fully elucidate the mechanisms of toxicity and to develop new treatment strategies through these mechanisms. Rodents (rats and mice) are particularly used to study liver toxicity in humans. Animal models currently in use include the ad libitum, Lieber–DeCarli diet model, Tsukamoto–French model, National Institute on Alcohol Abuse and Alcoholism (NIAAA) Model, and secondary intervention methods.^16^ Using an ideal model, it is possible to induce steatosis, disorders in fat metabolism, inflammation, neutrophil infiltration, and, when necessary, even fibrosis, cirrhosis, and cancer. When there are serious complications such as fibrosis, cirrhosis, and cancer, a second intervention method is often needed.^20^

### History of Experimental Models

The use of animals as models for the diagnosis and treatment of diseases in scientific studies dates back to ancient Greece. The first studies on this subject are observational, and their aim is to better understand human physiology. Galen of Pergamon, in the 2nd century B.C., studied neuroanatomy and the cardiovascular system using live animals. Aristotle carried out some studies on embryogenesis in chicks in the 4th century B.C.^21^ Flemish anatomist Vesalius (1514-1564) studied the similar and different features between human and animal anatomies and, as a surgeon, gave live slaughtering lessons on animals to medical students. In the 16th century, some physicians, including Servetus and Lusitano, concluded that the blood circulation in the body was divided into pulmonary and systemic. In the 17th century, William Harvey also conducted studies on animals. In 1628, Harvey published his revolutionary theory of circulation. With this theory, Harvey developed a radical approach to the functioning of the body.^22^ By the 20th century, the use of animal models was an issue that not everyone found ethically correct, but it became quite widespread in studies carried out to develop the diagnosis and treatment of diseases. As the studies increased, it was realized that genetic factors also had an impact on the study results, and the same species of animals began to be used in studies with the contributions of researchers such as William Castle, Halsey Bagg, Leonell Strong, and Clarence Little. As a result, different animal strains began to be created, and the differences between these strains revealed the importance of hereditary characteristics. This reflects the importance of species selection in animal modeling.^21^ Experimental studies are being conducted to understand the mechanisms underlying liver toxicity with numerous hepatotoxicity-inducing agents using different animal species.^23-28^ Many animal species have also been used to study alcohol-induced liver pathology.^13^ Although rodents (mice, rats, and hamsters) are primarily used, studies have also been conducted on minipigs and primates.^22^ However, the cost and study time make the use of primates prohibitive for most research laboratories. Therefore, rodents (rats and mice) remain the most commonly used animal model.^29^ Unlike humans, alcohol consumption in rodents does not constantly increase. The rate of ethanol breakdown in rodents is 5 times faster than in humans, and the increase in acetaldehyde concentration in the blood causes rodents to stop consuming alcohol.^30^ The basal metabolism of rodents is also generally faster than that of humans. Therefore, these differences between rodents and humans should be taken into consideration when determining the models to be selected.^31^

## Current In Vivo Experimental Models Used in Alcohol Toxicity

### Ad Libitum Model

The ad libitum model is one of the oldest methods known to be used in studies on the effects of alcohol on the liver in rodents.^32^ This method is the simplest alcohol feeding method to implement. There is only 1 tap from which rodents can drink, and from this tap, alcohol is given to rodents in desired concentrations that can be changed at any time. In addition to alcohol, the animals are given standard rodent food.^22,33^ In this way, the ad libitum model resembles the typical pattern of human behavior regarding voluntary alcohol consumption combined with a normal diet.^22^ In this model, the alcohol concentration added to the animals’ drinking water can be gradually increased with an appropriate diet.^33^ In animals using the ad libitum model, liver damage can be induced by increasing alanine aminotransferase (ALT) and aspartate aminotransferase (AST), and inflammation and hepatic steatosis can be generated, but this model is insufficient to cause more advanced fibrosis or cirrhosis.^33-35^ Since animals are not forced to drink alcohol with the ad libitum model, this model can be used to explore molecules and neurochemical pathways that contribute to alcohol abuse in studies on behavior and addiction in humans.^36,37^ Since there are low mortality rates when following an ad libitum diet, chronic alcohol feeding can be maintained for a long time with the use of this model. For example, Cook et al^38^ fed different mouse strains with 20% (w/v) ethanol using the ad libitum method from 8 weeks to 78 weeks. The ad libitum model has advantages as well as various limitations. Because rodents have a natural aversion to alcohol and metabolize alcohol faster than humans (~4-5 times higher than humans), their blood alcohol concentration (BAC) cannot reach levels high enough to reveal levels of liver damage.^22,31^ Since high alcohol concentrations cannot be achieved using the ad libitum model, some studies have been conducted on whether the damage to the liver from chronically administered alcohol can be enhanced by dietary changes such as increased fructose intake or fat consumption. Song et al^35^ evaluated whether a high-fructose diet changed the effects of chronic alcohol use in 6-week-old male C57BL/6J mice. They fed mice 60% fructose for 18 weeks and gave them 20% (v/v) ethanol as chronic alcohol. They followed an ad libitum diet for 9-18 weeks. As a result, they observed that high amounts of fructose and chronic alcohol use both individually cause fat accumulation in the liver and that their combined use has a synergistic effect, increasing liver damage but not fat accumulation and increasing serum ALT and AST levels. Duly et al^39^ designed a study in male C57BL6 mice to see how a high-fat diet may affect the damage to the liver that can be caused by alcohol. Mice were divided into 2 groups; 1 group was fed with a chow diet containing 12% of total calories from fat, and the other group was fed with an ad libitum diet containing 45% fat and 0.25% cholesterol for 12 weeks. In addition, half of both groups were given a 2 g/kg saline solution with a 30% ethanol concentration via gastric tube twice a week. As a result of the study, a synergistic increase in high-density lipoprotein-inducible liver damage, an increase in serum triglyceride levels and liver weights, and lipid accumulation in vesicles were observed with the addition of alcohol to the high-fat diet. Additionally, an increase in inflammatory response and pro-fibrogenic changes were observed, and it was shown that this model is a method that can be used for studies to examine the development of fibrosis.

The use of some chemicals, such as CCl_4_, phenobarbital, and diallyl disulfide, together with the ad libitum model can cause advanced damage to the liver, such as inflammation, fibrosis, and liver cancer, which are difficult to achieve only with the ad libitum method. Chae et al^40^ studied 20 adult female rats for 10 weeks and divided the rats into 3 groups. In the first group, 8 rats were fed only with the ad libitum method. In the second group, they fed 6 rats with 10% alcohol and 50% 1 mL/kg CCl_4_ twice a week by intragastric tube. In the third group, the rats were fed with 10% alcohol, 10% CCl_4_, and 0.1 mg/kg lipopolysaccharide (LPS) intraperitoneally for 2 weeks. As a result of the study, cirrhosis and fibrosis development were observed in the second and third groups, while no fibrotic change was observed in the second group. In another study, adult male Wistar rats were divided into 4 groups. The first group was given 25% (v/v) ethanol and 500 mg/L phenobarbital in drinking water as the only drinking water source; the other group was given only 25% ethanol in drinking water; 1 of the control groups was given only drinking water; and the other control group was given 500 mg/L phenobarbital in drinking water. As a result of the study, fatty liver disease was observed, but no fibrosis or hepatitis was observed.^41^

As a summary, the ad libitum method is an easy and repeatable method, although it is difficult to reach the desired concentrations. It is also suitable for secondary interventions that we will talk about in the following sections of the article.

### Lieber–DeCarli Model

Although the toxicity of alcohol to the liver is well known today, studies on this subject continue. Until the early 1960s, researchers thought that liver damage after alcohol consumption was a result of malnutrition and that alcohol alone did not pose a hepatotoxic risk.^32^ In the 1960s, Lieber et al^42^ added ethanol to the drinking water of mice and examined the liver, and they took the old studies one step further and measured the amount of ethanol in the blood of mice. But since the rodents showed a serious aversion to alcohol, no change was observed in the results, and the amount of ethanol in the blood was found to be at negligible levels. Therefore, it was not possible to cause liver toxicity with the amount of ethanol used in these experiments.^42-44^ Thereupon, Charles Lieber, in collaboration with Leonore M. DeCarli, developed a unique method to overcome rats’ aversion to alcohol. In this method, animals were given a completely liquid diet, and ethanol was given along with this liquid diet. While it was observed that the reluctance felt by the mice could be prevented if they were not given food or drink other than the liquid diet, daily ethanol intake could reach up to 12-18 g/kg. These amounts were almost 2-3 times the ethanol concentration that could be achieved in mice fed only ethanol.^45^ They thus concluded that alcohol alone is a pathological factor that can trigger liver disease.^45,46^ These researchers’ liquid diet formulas, which are now standard experimental models for studies and the nutrient content summarized in Table 1, were eventually named the Lieber-DeCarli ethanol (LDE) and Lieber-DeCarli control (LDC) diets.^45^

The LDE diet is an isocalorically regulated liquid diet in which some ingredients are modified to suit various groups and experimental objectives, but the overall caloric content of the diet (0.6-1.0 cal/mL) stays constant. In the LDE diet, casein constitutes 18% of the required calories and contains methionine and cysteine; dextrin constitutes 11%; maltose constitutes 47%; and fat constitutes 35% of the calories. Olive oil, safflower oil, and corn oil are generally used as fats. The diet is supported by essential vitamins such as A, D, E, K, B, minerals, and fiber content. The amount of ethanol to be used in the diet is gradually increased to cover 5 mg/dL or 36% of the calories in the diet.^46,47^ After starting the diet, 36% of the carbohydrate amount in the diet is isocalorically replaced by alcohol for 7-10 days. Alcohol concentration increases over time.^33^ If less than this amount of alcohol is consumed, the desired concentration in the blood cannot be reached, and consuming a higher amount of alcohol (more than 5 mg/dL) does not provide any more benefit for modeling.^46^

The application period of the LDE diet varies depending on the purpose of the study, which rodent is used, and gender.^37^ In rodents fed with this model for 4 weeks, a 6-fold increase in hepatic triglycerides, CYP2E1 induction, a significant increase in serum AST and ALT values, reactive oxygen species (ROS) production, mild steatosis, and infiltration of inflammatory cells occurred, whereas when the diet was continued for as long as 9 months, fibrotic changes other than steatosis occurred. No significant hepatic pathological changes were observed.^8,29,48,49^ In Xu et al’s^50^ study, the LDE diet was first applied for 5 days so that the mice could get used to the diet. After day 6, the LDE diet containing 5% ethanol was administered to mice for 10 days. On the 16th day, a single dose of 20% ethanol was administered to the mice (the amount of ethanol was adjusted to be isocaloric with dextrin and maltose), and the experiment was terminated after 9 hours. As a result of their studies, they observed an increase in ALT and AST values, fatty tissue in the hepatic tissue, an increase in ROS production, a decrease in glutathione levels, an increase in the levels of inflammation biomarkers tumor necrosis factor alpha (TNFα) and interleukin 6 (IL-6), and an increase in CYP2E1 activity in mice. Higher BACs are needed to cause greater damage from steatosis. These values cannot be achieved with the LDE diet.^50^

When using this model, a secondary hepatic stressor can be used to increase its effect. For example, successful results have been achieved with the use of hepatotoxins or viral proteins such as CCl_4_, a high-fat diet, acetaminophen, iron, or diethylnitrosamine (DEN).^51^ In Fujimoto et al’s^51^ study, female C57BL/6J mice were fed with an LDE diet with 2.5% (v/v) of total calories being ethanol for 8 weeks. One mL/kg of CCl_4 _administered intraperitoneally twice a week. It was observed that mice experienced an increase in serum transaminase levels, fat accumulation in the liver, increased lipid peroxidation and pro-inflammatory response, an increase in stellate cells in the liver, expansion of (F4/80-positive) Kupffer cells, and fibrosis. In another study, Rafacho et al^52^ administered 24 mg/kg DEN intraperitoneally to 14-day-old C57BL/6 mice for 9 weeks. At 8 weeks of age, mice were fed either the LDE control diet or the ethanol diet, with 27% of total calories being ethanol. As a result of the study, steatosis was observed in mice administered the ethanol diet + DEN, and a significant increase in preneoplastic lesions, which are important markers indicating the presence of inflammation, was observed.^52^

The LDE diet is useful for monitoring the early stages of alcoholic liver disease in chronically fed rats and the metabolic changes that alcohol induces in the liver and other organs.^46^ However, this diet method is not physiological, as animals are forced to consume alcohol every time they are hungry and thirsty. In addition, one of the serious limitations of the LDE model is that it cannot create fibrosis or very little inflammation.^45^

### Tsukamoto–French Intragastric Infusion Model

Models in which alcohol is given orally to animals, such as the ad libitum and LDE models that we mentioned, cause ethanol-related damage to the liver. Although there are effective methods for this, these models have serious limitations. The BAC value obtained with orally administered alcohol is lower than the BAC value in humans. In addition, although steatosis can be created with these methods, fibrosis or cirrhosis cannot be created without a secondary stress factor.^22,33^ In order to prevent the BAC levels that can be achieved with these models, in which alcohol is administered orally, from not reaching the BAC level that could cause damage to humans, the Tsukomoto–French intragastric infusion model, an enteral feeding model, was developed.^53,54^ The main purpose of developing this model is to overcome the natural aversion of rodents to alcohol and to cause liver damage by consuming high doses of alcohol in rodents that do not voluntarily consume sufficient amounts of alcohol.^54^ In this model, alcohol and nutrients are injected directly using an intragastric cannula implanted in the animals’ stomachs, with ethanol accounting for 49% of total calories.^55^ A significant increase in ALT and AST values occurred in animals to which the diet was applied with a regular infusion of this model (22-35 g/kg/day) for one month. However, the BAC value increased to an average of 300 mg/dL, resulting in hepatic steatosis and focal necrosis.^56^ Ethanol administered using the Tsukomoto–French model can cause steatosis, apoptosis, fibrosis, necrosis, and inflammation, which are very similar to alcoholic liver disease in humans.^57^ The Tsukomoto–French model also allows the dietary content to be adjusted as desired to generate the desired liver damage pattern. With the addition of polyunsaturated fatty acids to the diet (25% of total calories), steatohepatitis in rodents was further increased, resulting in liver fibrosis in animals after 4 months.^58^ In the study conducted by Tsukomoto et al,^58^ liquid diet infusion was administered to 17 pairs of male Wistar rodents, with 25% of the total calories being fat + ethanol or isocaloric dextrose given via gastrostomy cannulas. In order to maintain toxicity, the amount of ethanol given was increased from 32% to 47% of the total calories. As a result of the study, moderate or severe fat filtration was observed in all rats. Necrosis and fibrosis, along with cell infiltration, were observed in 14 of the rats. In another study, Tsukomoto et al,^59^ observed whether the severity of alcoholic liver fibrosis could be increased by supplementing the diet with iron. Iron was infused with or without ethanol via intragastric infusion for 16 weeks. By adding carbonyl iron (0.25% weight/volume) to the diet as an iron supplement, fibrosis developed in 60% of the animals and cirrhosis developed in 17%. A new hybrid model based on the original alcohol catheter model was created in 2015 by Tsukamoto et al. A gavage catheter was placed for the purpose of infusing ethanol into the animals after they had been fed a diet high in saturated fat and cholesterol for 2 weeks. The 8-week model period saw a progressive increase in ethanol intake to 27 g/kg per day. After the second week, alcohol intake was limited to once per week (4-5 g/kg). Among these, repeated administration of ethanol to animals triggered the transition from chronic alcoholic steatohepatitis to acute alcoholic hepatitis. This model demonstrated for the first time the clinical features of alcoholic hepatitis, such as hypoalbuminemia and hyperbilirubinemia.^60^ The Tsukomoto–French model is a model that induces liver damage similar to alcoholic liver disease in humans, as it can also cause advanced steatosis, fibrosis, and even cirrhosis, accompanied by immune cell filtration and focal necrosis.^22,58^ Serious liver damage can be caused with the Tsukomoto–French model, but like other methods, this method has some disadvantages. The most important disadvantage is that the intragastric catheters to be used in animals require surgical placement.^57^ In addition, the method requires expensive materials, and serious care is required for the animals to prevent infection, as it may cause increased mortality in the animals after the operation.^22,54,55,57^

### National Institute on Alcohol Abuse and Alcoholism Model (Gao Binge Model)

The NIAAA model, a modification of the LDE diet, was developed by Bin GAO and his team in 2013.^37,61^ This model mimics an acute or chronic alcoholic liver injury in patients. According to the application protocol of the model, the LDE diet is applied to animals for 10 days, containing 5% v/v ethanol. Then, on the 11th day, a single dose of 5% g/kg ethanol is given, and the animals are euthanized 9 hours later. As a result of the application of this model, an increase in ALT/AST values and neutrophil infiltration are observed.^61^ The effects of alcohol on liver damage were tested as a result of changes in the NIAAA model with different modifications. As an example of these modifications, in Kirpich et al’s^62^ study, an ethanol-containing LDE diet was used using C57BL/6N mice. This diet is enriched with corn oil as an unsaturated fat and beef tallow as a saturated fatty acid. As a result of the study, it was observed that alcohol is an important factor in liver damage; saturated fatty acids (corn oil) increase intestinal permeability, and alcohol further exacerbates this effect. Additionally, an increase in the amount of endotoxin in the blood and upregulation of Toll-like receptors in the liver have been observed with unsaturated fatty acids (beef tallow) and alcohol. In another example of modification, Aroor et al used chronic LDE feeding with a single dose of high ethanol (5 g/kg) or a repeated intragastric infusion of ethanol (5 g/kg, 32% v/v, 3 doses, 12 hours apart) has been administered. As a result of the study, it was observed that liver damage increased. The biggest advantage that can be achieved by making this modification is that neutrophil infiltration can be increased. The highest BAC level that can be achieved using the NIAAA model is 400 mg/dL.^57^ Higher BAC values can be achieved with this model compared to the LDE diet. It may also create more steatosis, and additionally, neutrophil infiltration may be increased. Severe steatohepatitis can occur with long-term chronic feeding and the further administration of multiple high doses of ethanol. Additionally, the NIAAA model is efficient in terms of time and cost.^45^ Like every model, the NIAAA model has its disadvantages. Animals to which the model is applied experience excessive weight loss and high mortality.^37^ The NIAAA model mimics alcoholic liver damage in the form of binge drinking in chronic alcohol abusers.^63^

### Secondary Intervention Methods

Steatosis can be caused in rodents with the use of the models we mentioned and appropriate amounts of alcohol and calorie regulation. However, when it comes to modeling cirrhosis or fibrosis caused by alcohol in the liver, a second intervention method is needed.^20^ The second most well-known intervention models are: dietary changes (eating a high-fat diet), xenobiotics such as CCl_4_, hepatotoxic substances such as DEN, viral infection, or genetic changes.^22,57^

### Changes That Can Be Made in the Diet

Among the changes that can be made in the diet, the most common is the environmental secondary intervention method.^57^ As we mentioned before, it is known that a high-fat diet increases the damage caused by alcohol to the liver.^20^ The most important effect of feeding a high-fat diet is the induction of the CYP2E1 enzyme.^64^ In the diets used, the amounts of carbohydrates, fats, and ethanol are adjusted isocalorically; therefore, by increasing the amount of unsaturated fatty acids in the diet, the amount of carbohydrates to be taken will be reduced. Since isocaloric nutrition is generally used, high levels of fat reciprocally lead to lower carbohydrate content. This low-carbohydrate–high-fat diet induces CYP2E1 and subsequent oxidant stress and alcoholic liver damage. In a study, while steatosis could be developed only with a high-fat diet, CYP2E1 induction, an increase in serum ALT value, and hepatic necrosis were observed with a carbohydrate-free diet.^65^ In the study conducted by Tsukamoto et al,^58^ it was shown that fibrosis was created by increasing the amount of fatty acid and ethanol given to animals isocalorically. Eating a high-fat diet can also cause obesity, insulin resistance, and nonalcoholic fatty liver disease. It also causes an increase in endoplasmic reticulum (ER) stress.^20^ In a study by Chang et al,^66^ animals were fed a high-fat diet for 3 days and 3 months. Then, a single high dose (31.25% in water after 3 days and 5% g/kg in 53% water after 3 months of application) of ethanol and isocaloric dextrose–maltose was applied. According to the data obtained from the study, the most important result is neutrophil infiltration. While for more severe steatohepatitis, an increase in hepatic macrophages and ALT and AST values was observed after 3 months, a decrease in hepatic macrophages was observed after 3 days of application.

Furthermore, the liver is crucial for preserving the body’s iron homeostasis.^67^ In the model where iron and alcohol are taken together, iron catalyzes and promotes liver oxidative stress and damage. In the study conducted by Sadrzadeh et al,^68^ it was shown that hepatic free iron content, lipid peroxidation, and fat accumulation decreased in ethanol-fed animals by using an iron chelator. In the Tsukomoto–French model, as a result of the addition of carbonyl iron to the high-fat diet (25% of total calories), serum ALT and AST levels increased to 2-3 times those of rats fed a normal diet, which led to the development of fatty liver as well as liver fibrosis and even cirrhosis in some animals.^59^

Other changes that can be made in the diet may include following a diet deficient in zinc, folate, and choline.^69-71^ Applying a choline-deficient diet to animals for 1-2 weeks causes a decrease in lipotropes that protect the liver from becoming fatty, resulting in hepatic steatosis. Hepatitis, fibrosis, cirrhosis, and even hepatic carcinoma may occur in animals fed a choline-deficient diet for more than 3 months.^13^ Choline and folate are necessary for methionine synthesis. It should be noted that the degradation of methionine generally occurs in the liver with the enzyme methionine adenosyltransferase and the formation of S-adenosylmethionine (SAM). Likewise, deficiency of the methionine adenosyltransferase enzyme also causes a decrease in lipotropes. SAM is a methyl donor and glutathione precursor. Liver enlargement occurs as a result of a decrease in the amounts of SAM and glutathione. Additionally, the liver becomes more susceptible to injury, and the likelihood of spontaneous steatohepatitis increases. A change in methionine metabolism may also affect the stellate and Kupffer cells in the liver and have serious effects, including liver cancer.^72^ Therefore, choline and folate can be used as secondary intervention methods.

### Agonistic/Xenobiotic/Pharmacological Secondary Intervention Methods

The exogenous stimuli LPS, CCl_4_, acetaldehyde compounds, acetaminophen (APAP), and DEN are frequently used to cause the secondary hit.^57^ According to a study, LPS (10 mg/kg) was injected intravenously after rats were given an LDE diet for 10 weeks. In animals fed chronic ethanol combined with low-dose LPS, neutrophil infiltration was observed, necrosis was formed in liver cells, inflammation was induced, and serum AST levels increased.^73^ The advantage of the ethanol + LPS model is that the method is easy and can be applied in many laboratories.^57^

Feeding animals with a high-fat diet, as well as xenobiotics such as CCl_4_ and pyrrosol, may induce CYP2E1. Like alcohol or LPS, they can cause cell damage and even fibrosis. APAP is one of these xenobiotics. Alcoholic people are more sensitive to APAP.^20^ In a study conducted on mice, the susceptibility of the livers of animals chronically fed ethanol to APAP-induced damage was increased. Chronic ethanol administration is thought to increase APAP hepatotoxicity through glutathione depletion and the induction of CYP2E1.^74^ In another study, APAP was administered intraperitoneally to mice fed an LDE diet containing ethanol, and a significant increase in ALT and AST levels was observed in these animals.^75^

One of the most effective models that can be applied to create fibrosis in the liver is the ethanol + CCl_4_ model.^57^ Some studies have been conducted on the effects of CCl_4_. In a study conducted by McCuskey et al^74^ on rodents, CCl_4_ was administered to rodents via inhalation 5 days a week, 6 hours a day, for 10 weeks, and it was shown that fibrosis was formed after 5 weeks of administration, while cirrhosis was caused after 10 weeks of administration. In a study, the model created by injecting CCl_4_ (0.5 μL/kg, every 3 days) into the peritoneal cavity of mice along with the LDE diet for 8 weeks was similar to alcohol-induced cirrhosis in humans.^76^ In the Brol et al^77^ study, the animals were given drinking water with 4% ethanol in the first week, 8% ethanol in the second week, and 16% ethanol in the remaining weeks, for a total of 7 weeks. Subsequently, CCl_4_ was administered to the animals via inhalation. In the study, while fibrosis could be created after 4 weeks of application, it was observed that proinflammatory responses increased significantly after 7 weeks. There is also a study showing that CCl_4_ causes liver cancer. Animals were injected intraperitoneally with CCl_4_ twice a week for 28 weeks, and 4% ethanol was administered along with the LDE diet for the last 10 weeks. As a result of the study, steatosis, fibrosis, increased inflammation, ballooning, and tumor nodules as a result of degeneration of hepatocytes were observed. This study showed that alcohol-related liver cancer can be caused by adding ethanol to the diet after liver fibrosis has been established.^78^ The ethanol + CCl_4_ model is a very valuable model for creating fibrosis. Moreover, although it is a simple and cheap method, it is a time-consuming model.^57^

Nitrosamines are toxic substances for both animals and humans. In low doses, they cause serious liver damage through oral or parenteral administration. The damage caused by *N*-nitrosodiethylamine (DEN) in the liver notably includes neutrophil infiltration, necrosis, fibrosis, and the potential formation of liver cancer. The hepatocarcinogenicity of DEN makes it intriguing for use as a secondary intervention method.^79^ For this purpose, mice are frequently used to reproduce the DEN hepatocellular carcinoma (HCC) model. In a study conducted by Ambade et al,^80^ mice were fed with the LDE diet, and intraperitoneal DEN was administered (75 mg/kg for the first 3 weeks, followed by 100 mg/kg for the next 3 weeks) to induce hepatocellular cancer. Starting from the seventh week, animals were continued on a 4% LDE diet for 7 weeks before euthanasia was applied. The study observed an increase in inflammation and fibrosis in the liver, which are associated with hepatocarcinogenesis in humans. In another study, Sun et al^81^ injected DEN intraperitoneally at an initial dose of 50 mg/kg, followed by 25 mg/kg DEN injections every 4 weeks. After the first DEN injection, alcohol was added to the drinking water of the mice (2% for the first 3 days, 4% for the next 3 days, 8% for the next 10 days, 12% for the following 9 days, and finally 16% for the rest of the experiment) at isocaloric intervals. Subsequently, the necessary samples were collected. Sun et al observed a decrease in the survival rate of animals, an increase in liver infections, liver damage, fibrosis, and, most importantly, the contribution of DEN to liver cancer as a result of the combined use of alcohol and DEN. In a different study, mice were given an intraperitoneal DEN injection (10 mg/kg) at the age of two weeks, and then at 3 months, they were placed on a 3-7-week LDE diet (including 4.8% alcohol). In this paradigm, mice were given ethanol and DEN to produce obvious surface tumors.^82^ The ethanol + DEN concept is affordable and easy to apply. It is also one of the most frequently utilized models for HCC since it histologically and genetically resembles HCC caused by alcohol.^57^

### Genetic Secondary Intervention

There are 2 aspects of using genetics as a second intervention method. One of these is increasing the function of pathogenic genes that cause liver damage.^57^ For example, it may be possible to increase the function of the CYP2E1 enzyme, which is one of the most important enzymes responsible for the metabolism of alcohol in the liver.^20^ Morgan et al^83^ studied alcoholic liver disease by generating CYP2E1 transgenic mice and observed that more liver damage occurred in alcohol-fed transgenic mice. The second method that can be used is to suppress the protective genes that protect the liver from the effects of alcohol.^57^ Examples of these genes include the suppression of nuclear factor-erythroid 2-related factor 2 (Nrf2), superoxide dismutase, and IL-6 genes.^84-86^ Nuclear factor-erythroid 2-related factor 2 is a gene responsible for protecting our cells from xenobiotics and oxidative stress. Increased oxidative stress is important for alcohol-related liver disease. Lamle et al’s^84^ study observed that Nrf2(−/–) loss causes problems in the detoxification of acetaldehyde, increases fat in the liver, causes structural and functional changes in mitochondria, increases inflammation mediated by Kupffer cells, and eventually liver failure occurs due to the accumulation of damage in the liver. The female gender is the best example of a genetic model that does not need to be genetically altered. Liver illness caused by alcoholism is more common in women. In a study by Liimuro et al,^87^ using an intragastric ethanol infusion model, it was shown that steatosis, inflammation, and necrosis developed faster and more severely in female mice.

### Viral Secondary Response

Hepatitis B (HBV) and hepatitis C (HCV) are important global risks for HCC.^88^ Alcohol consumption and HCV have a synergistic toxic effect on the liver. Their combined use increases the risk of liver diseases.^89^ According to studies, 30%-40% of alcoholic liver patients have HCV, while 70% of HCV patients are known to consume excessive alcohol. The relationship between these two diseases has an impact on genetic predisposition. It also causes oxidative stress. It is possible that ROS overproduction, TNFα and TGF-β expression, changes in the immune response, increased lipid peroxidation in the liver, and synergistic effects on the mechanisms that cause cancer.^90^ Perlemuter et al^90^ used 10-month-old male HCV core transgenic mice (strain C57BL/6N) in their study. To observe acute toxicity, ethanol was administered by gastric intubation at a level of 25% ethanol concentration in the water (2.5 g/kg). For chronic toxicity, an ad libitum diet was used. They started the amount of ethanol administered in drinking water at 5% and gradually increased it to 20% within 2 weeks. As a result of the study, an additive effect on lipid peroxidation in the liver was observed. According to research, liver fibrosis and progression to HCC (to the next stage of cirrhosis) develop more rapidly in HCV-positive patients who consume alcohol than in those who do not drink alcohol.

Hepatitis B is a risk factor for hepatocellular carcinoma and death in alcoholic liver patients. Liu et al^91^ fed mice with 5% ethanol and a sufficient LDE diet for 4 weeks and increased the ethanol rate to 7% ethanol for the next 4 weeks. At the end of the study, it was observed that HBV and ethanol induced abnormal hepatic lipid metabolism with a synergistic effect in mice and led to fatty liver disease.

## Conclusion

Alcoholism is now recognized as a major global health issue. The health and socioeconomic consequences of alcohol consumption represent a heavy burden worldwide. Although significant progress has been made in better understanding the mechanisms and pathology of alcohol-related liver diseases, many features of these diseases remain unknown. Various experimental models have been created to investigate the mechanisms of alcohol-related liver diseases. These models have different advantages and disadvantages (Table 2). The disadvantages of the ad libitum model, which can be used because it is easy and reproducible, are that it cannot reach high concentrations and that it cannot prevent reluctance in rodents. The Lieber–DeCarli diet was developed to prevent this reluctance in animals. In this model, the disadvantages are that the animals are forced to drink alcohol every time they feel hungry or thirsty and that they cannot cause more serious liver damage, such as fibrosis. The desired blood concentrations cannot be achieved by oral administration of alcohol. To avoid this problem, the Tsukomoto–French intragastric infusion model was developed. The biggest advantage of this model is that high concentrations can be reached. Despite its costliness, the model is associated with elevated animal mortality rates and necessitates meticulous care to reduce the risk of infection. The NIAAA model, developed as a modification of the LDE diet, is similar to the drinking patterns of people who use chronic alcohol or consume excessive alcohol. In this model, by giving one or multiple doses of ethanol in addition to the LDE diet, higher blood concentrations can be achieved compared to the LDE diet. However, this model causes weight loss in animals and shows high mortality. Since all these models do not cause serious liver damage such as cirrhosis, fibrosis, or cancer, the use of only these models is insufficient. For this reason, more serious damage can be caused by additional secondary intervention methods.

Although there are differences in the degree and stages of alcohol-related liver damage between rats, mice, and humans, rodent models are currently extremely useful in improving our knowledge of alcohol-related liver diseases. We hope that in the future, an ideal model in rodents will be able to effectively mimic step-by-step how alcohol causes liver damage in humans.

## Figures and Tables

**Table 2.** Comparison of Experimental Models

Models	Advantages	Disadvantages
Ad libitum model	Easy to perform. Minimal elevation of ALT and mild steatosis. Short-or long-term feeding with no mortality rate.	Insufficient for fibrosis or cirrhosis. Desired BAC levels cannot be achieved.
Lieber–DeCarli model	Easy to perform. Increase in hepatic triglycerides. CYP2E1 induction, marked elevation of AST and ALT. Reactive oxygen species production. mild steatosis. infiltration of inflammatory cells can be observed.	It is not physiological. No liver fibrosis.
The Tsukamoto–French intragastric infusion model	Marked elevation of AST and ALT and steatosis.	Difficult to perform. Requirement for intensive medical care. Expensive materials required. Mild liver fibrosis. Long-term feeding with a high mortality rate.
The NIAAA Model	Cost and time efficient. High blood alcohol levels. Liver injury. Inflammation. Fatty liver.	Animals to which the model is applied experience high weight loss and the mortality of the model is also high.
Secondary intervention methods	Simple and cheap. Moderate to significant elevation of serum ALT, AST, and liver. inflammation dependent on second hit. Liver fibrosis. Liver cancer.	Time consuming. Toxic components.

ALT, alanine aminotransferase; AST, aspartate aminotransferase; BAC, blood alcohol concentration.

**Table 1.** Nutrients in a Standard Lieber–DeCarli Diet and Their Percentages in the Diet

Content	Carbohydrate		Protein	Lipid	Ethanol
	Dextrin	Maltose	Casein (Contains Cysteine, Methionine)	Olive Oil, Safflower Oil, Corn Oil	
Percentage of calories in diet (%)	11	47	18	35	36
